# Hyperbaric oxygen therapy for the treatment of hypoxic/ischemic injury upon perinatal asphyxia—are we there yet?

**DOI:** 10.3389/fneur.2024.1386695

**Published:** 2024-04-15

**Authors:** Damian Mielecki, Jakub Godlewski, Elzbieta Salinska

**Affiliations:** ^1^Department of Neurochemistry, Mossakowski Medical Research Institute, Polish Academy of Sciences, Warsaw, Poland; ^2^NeuroOncology Laboratory, Mossakowski Medical Research Institute, Polish Academy of Sciences, Warsaw, Poland

**Keywords:** apoptosis, brain, hyperbaric oxygen therapy, microRNA, oxidative stress, perinatal hypoxia-ischemia

## Abstract

Birth asphyxia and its main sequel, hypoxic-ischemic encephalopathy, are one of the leading causes of children’s deaths worldwide and can potentially worsen the quality of life in subsequent years. Despite extensive research efforts, efficient therapy against the consequences of hypoxia-ischemia occurring in the perinatal period of life is still lacking. The use of hyperbaric oxygen, improving such vital consequences of birth asphyxia as lowered partial oxygen pressure in tissue, apoptosis of neuronal cells, and impaired angiogenesis, is a promising approach. This review focused on the selected aspects of mainly experimental hyperbaric oxygen therapy. The therapeutic window for the treatment of perinatal asphyxia is very narrow, but administering hyperbaric oxygen within those days improves outcomes. Several miRNAs (e.g., mir-107) mediate the therapeutic effect of hyperbaric oxygen by modulating the Wnt pathway, inhibiting apoptosis, increasing angiogenesis, or inducing neural stem cells. Combining hyperbaric oxygen therapy with drugs, such as memantine or ephedrine, produced promising results. A separate aspect is the use of preconditioning with hyperbaric oxygen. Overall, preliminary clinical trials with hyperbaric oxygen therapy used in perinatal asphyxia give auspicious results.

## Introduction

Perinatal asphyxia is a pathological condition caused by impaired blood flow to organs (due to hypoxia-ischemia), before, during, or after birth. Hypoxic–ischemic encephalopathy (HIE), occurring upon hypoxia-ischemia (HI), is a major contributor to global child mortality and morbidity. The prevalence of birth asphyxia is approximately 2–6 per 1,000 live-born infants. As many as 15–25% of them die, while 25% are diagnosed with permanent neurological damage (1).

The mechanism underlying perinatal brain injury after HI originates as an interruption of placental blood flow followed by impaired gas exchange, causing deficits in oxygen and metabolic substrate delivery to the child’s central nervous system. These irregularities lead to energy failure characterized by decreased ATP production and an increase in lactate concentration, resulting in systemic acidosis. Energy deficit initiates a cascade of intracellular events leading to neuronal cell death. The first stage is the depolarization of neurons and glia and the release of excitatory amino acids, mainly glutamate. Activation of NMDA receptors, the critical group of ionotropic glutamate receptors, triggers an excitotoxic cascade (excessive exposure to glutamate) that generates reactive oxygen (ROS) and subsequent oxidative cell damage and initiates inflammation and apoptotic processes. The composition and activity of immature brain NMDA receptors differ from those identified in the adult brain, and their sensitivity to HI conditions increases excitotoxic damage (2). These intracellular mechanisms can continue their destructive activity resulting in myelin deficits, reduced plasticity, and delayed neuronal death. This process can continue for days or even years after the initial HI insult.

Therapeutic hypothermia initiated within a short time after birth was, for a long time, the only clinically available intervention for moderate and severe cases of HI. The treatment is often supported by medication to regulate blood pressure and control seizures, and dialysis to support the kidneys. However, these modalities are not efficacious enough to significantly improve the survival outcome and prevent infant brain damage. Moreover, it was reported that 64%, of children with mild HIE treated with hypothermia still developed moderate HIE (3). Therefore, many experimental therapies are still under investigation. Hypothermia is often combined with inhibitors of calcium channels and ionotropic glutamate receptors (topiramate, memantine, magnesium sulfate, 50% xenon), erythropoietin, or phenobarbital (4, 5). Hyperbaric oxygen therapy (HBOT) is one of the propositions for HI treatment (6).

**Figure 1:**
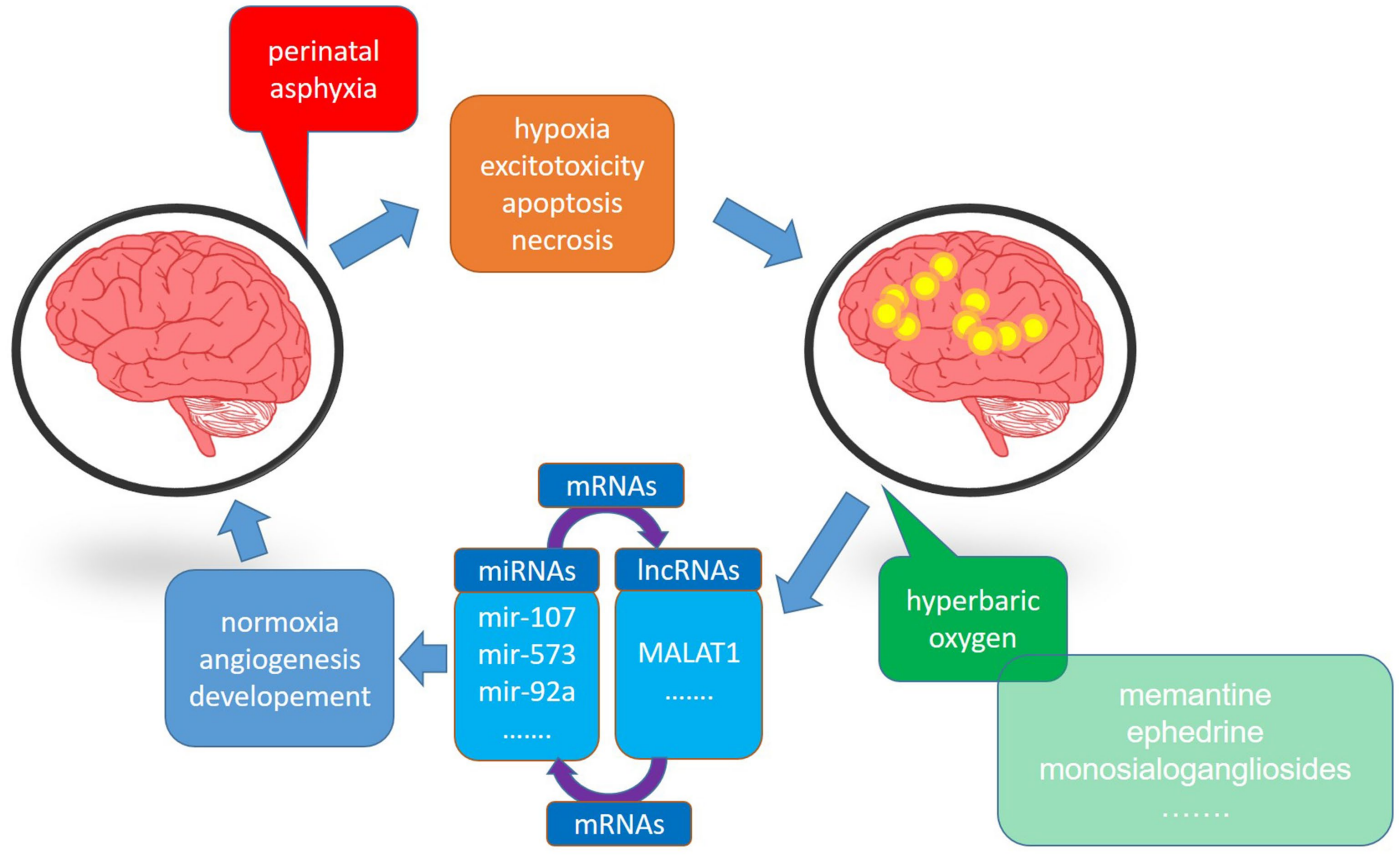
The schematic diagram showing the impact of hyperbaric oxygen therapy on the deleterious effect of hypoxia-ischemia (HI) on the brain. HI induces energy deficiency, apoptosis, necrosis, and loss of brain functions. On the other hand, hyperbaric oxygen, supplied in an appropriate therapeutic window, restores normal cellular functioning, significantly reducing brain injury. Both processes exert their activities through miRNAs and lncRNAs.

The efficacy of HBOT proposed in the 1960s as an experimental treatment of neonatal HI, was controversial due to the inconsistency of outcome benefits in early trials (7–9). Since then, however, HBOT has demonstrated effectiveness in perinatal asphyxia at <3.0 ATA not only due to the neuroprotective effect but also the apparent lack of side effects such as retinopathy (10–12). The neuroprotective mechanism of HBOT is related to an increase in the oxygen reservoir at the cellular level and triggering/supporting intracellular defense mechanisms (Figure 1). However, some HBOT-mediated neuroprotective effects in neonates are controversial, as results from different groups are inconsistent. This article will thus focus on intracellular HBO-activated defense mechanisms. The bulk of experimental data comes from the most universally accepted model of neonatal hypoxia-ischemia according to the Rice-Vannucci procedure in 7-day-old rats (13). Furthermore, we will discuss data from clinical trials where HBO therapy was used to treat neonatal patients and the current clinical use of HBOT in hypoxic–ischemic neonates.

## The effects of HBO treatment in experimental hypoxia-ischemia

### Therapeutic window

The established optimal therapeutic window for HBO intervention in neonatal hypoxia-ischemia is 6 h from the HI occurrence, and experimental HI in neonatal rats fits into this time frame. Within this therapeutic window, HBO was highly effective in reducing brain damage, predominantly neuronal loss in HI-sensitive regions, such as the CA1 area of the hippocampus and cerebral cortex (14–16).

Wang et al. showed that HBO treatment, administered for 60 min and repeated for 7 consecutive days, could be delayed by 12 h from the time of HI (14). HBO therapy started 48 h after HI did not instigate brain protection (17), although it was also shown that 60 min of HBO treatment started 24 h and repeated for 14 days might improve brain functional outcome and reduce neuronal death in neonatal rats in the model of intrauterine HI (18). Unfortunately, reports systematically determining the maximum duration of HBOT use are lacking. However, depending on the HBO treatment protocol, regarding the starting point of the treatment, the duration of a single session, and the number of treatments, results suggest that the time window can be expanded beyond the assumed 6 h.

Taking into account the limited time for effective intervention after perinatal asphyxia and anticipating complications that may result in HI, scientists look for alternative methods by which brain damage can be prevented, in addition to the already existing effective therapy of HI. These considerations prompted the concept of preconditioning: a treatment that uses noxious stimulus short of triggering the damage but strong enough to instigate defense mechanisms in the tissue poised to be exposed to damaging factors. Murry and colleagues first introduced the concept, documenting a protective effect of brief ischemic episodes applied before subsequent sustained heart ischemia in dogs (19). Since then, ischemic preconditioning has been thoroughly studied in different cases of heart and brain ischemias (20). HBO preconditioning (HBO-PC) also became the subject of research and its beneficial effects in neuroprotection were reported (21, 22). However, only three studies have validated the efficacy of HBO-PC in preventing brain injury after HI. A single session of HBO-PC, conducted at 2.5 ATA for 2.5 h (23, 24) as well as three days of sessions at 2.5 ATA for 2.5 h each (25) significantly reduced brain damage, oxidative stress, and apoptosis after neonatal HI in a rat model. Despite the authors’ claims of the safety and efficacy of HBO-PC for neonatal HI, its introduction into clinical practice is problematic as the occurrence of perinatal hypoxia is usually unpredictable, and in such cases, obstetricians usually deal with them more conventionally.

### Oxidative stress inhibition

One of the major issues related to perinatal HI brain injury is oxidative stress resulting from impaired mitochondrial functioning that leads to the overproduction of reactive oxygen species (ROS), such as singlet oxygen, hydroxyl radical, superoxide anion, hydrogen peroxide, and nitric oxide. Excitotoxicity caused by excessive release of excitatory neurotransmitters such as glutamate sets the process in motion. Mechanistically, in response to excitatory postsynaptic currents, mitochondria uptake excess cytosolic calcium, producing high ROS concentrations during disturbed mitochondrial respiration (26). Neuronal nitric oxide synthase (nNOS) can have a deleterious effect after HI, leading to the additional accumulation additional amount of reactive nitrogen species (RNS), e.g., peroxynitrite (27, 28). Three main antioxidant enzymes, superoxide dismutase (SOD), glutathione peroxidase (GPx) supported by glutathione, and catalase are mobilized to counteract the toxic effects and maintain the delicate balance of ROS in neurons (29).

An interesting review by Schottlender et al. summarized the effects of HBO treatment on mitochondrial function and oxidative stress, comparing protocols with varying oxygen pressure and time, and exposure frequency in different pathologies and healthy objects (30). In conclusion, a short HBO treatment (1–5 consecutive sessions) reduced mitochondrial function, while long expositions (20–60 consecutive sessions) improved mitochondrial parameters significantly. However, reports on the effect of HBOT used in experimental HI are not included and generally are sparse.

Few available publications brought valuable information on the effect of HBO therapy in experimental HI on the development of oxidative stress in neonatal rat brains. Several sessions of HBOT started up to 6 h after HI significantly reduced ROS levels in the brain (31). Increased SOD concentration measured in hippocampal homogenates derived from neonatal hypoxic–ischemic rats subjected to HBOT compared to the HI group was reported by Chen et al. (32). Moreover, the concentration of malondialdehyde (MDA), one of the critical end-products of lipid peroxidation, was significantly lower than in untreated animals.

Interestingly, Zhao et al. report increased glutamate accumulation and glutamate-induced oxidative stress in healthy rat puppies exposed to hyperoxia, accompanied by a decreased glutamate transporters expression (33). However, HBOT performed on ischemic adult rats significantly reduced glutamate release and ROS formation (34). Moreover, experimental data showed that SOD expression and anti-apoptotic Bcl-2 significantly increased in the brains of healthy gerbils subjected to five sessions of HBOT at 2 ATA (35), indicating different effects of HBO treatment depending on experimental conditions. Moreover, these data also suggested that the immature brain in HI conditions may respond to the HBOT differently than the one not exposed.

Barely two publications reported the effect on oxidative stress defense factors measured in the blood of neonates subjected to HBOT. Zhou et al. assessed the effect of HBO applied at different pressures (1.4–1.6 ATA, repeated for seven days) on changes in peroxidation and antioxidant levels in the serum of 60 neonates with HIE (36). SOD level was significantly higher and the serum concentration of MDA was significantly lower compared to the control group. Excessive activity of nitric oxide synthase (NOS) and nitric oxide (NO), apparent after HI and causing similar damage to ROS, was also significantly lower in examined serum samples of neonates.

Some authors (37) presenting the results of blood tests of 14 asphyxiated full-term newborns treated with HBO, reported no significant changes in SOD and catalase content; however, they noted a significant increase in the activity of GPx and reduction in ROS content.

The information cited above indicates that HBOT used in HI significantly stimulates antioxidant defense, which makes it an important element in stopping the development of oxidative stress and brain damage.

### Apoptosis inhibition

Apoptosis is a critical pathway of neuronal cell death after HI and many studies have aimed to prevent or reduce the development of apoptotic processes after HI.

The HBOT significantly reduced the percentage of apoptotic cells in most infant rat brain regions sensitive to HI, such as the hippocampus and cortex (31, 38, 39). Moreover, the HBO treatment 60 min after HI significantly reduced the level and activity of the pro-apoptotic caspase-3 (38, 39). Calvert et al. reported a significant amount of cleaved poly (ADP-ribose) polymerase (PARP) a caspase-3 substrate, which is considered to be a hallmark of apoptosis, in the hippocampus and cortex upon HI; however, HBO treatment significantly decreased cleaved PARP content in both regions (38).

Mitochondria release apoptosis-inducing factor (AIF), an essential element of caspase-independent apoptosis, in response to HI; however, the HBO treatment significantly attenuated such mitochondrial release and inhibited the caspase-independent neuronal cell death (39). It was also shown that HBO-PC significantly reduced the activity of caspase-3 and caspase-9 in the cortex and hippocampus compared to untreated animals in the neonatal HI rat model (23).

Hyperoxia elicits injury to premature lungs and retina and there is an opinion that it may cause brain damage if used in newborns; however, data presented above suggest that HBO treatment may effectively decrease HI-induced apoptosis, thus preventing brain damage. This effect of compressed oxygen may be beneficial only in hypoxic–ischemic conditions.

### Neural stem cell activation

The discovery of neural stem cells in the mammalian brain suggested regenerative potential for areas affected by HI injury, opening up new therapeutic possibilities. The HI significantly increased the number of neural stem/progenitor cells in the subventricular zone of 7-day-old rat puppies subjected to experimental birth asphyxia (40). As HI occurs during intensive brain growth and neurogenesis, even increased by HI conditions neural cell proliferation is insufficient to repair any damage and restore development completely. Thus, amplifying such a response could be a valid therapeutic approach. Of note, HBO enhanced the proliferation of neural stem cells in the subventricular zone of neonatal rats suffering from hypoxic–ischemic brain damage (41). Moreover, HBOT applied to HI neonatal rats significantly increased the levels of Wnt-3, the protein whose signaling pathway is associated with neurogenesis in neural stem cells. Similarly arose levels of nestin, the protein expressed in dividing cells during the early stages of the central nervous system development (42). These findings were thus in accord with previous reports on HBOT-mediated significant reduction of the ischemia-induced downregulation of neurotrophin-3, which promotes the survival of progenitors and their differentiation into neurons in the forebrain of rats (43).

### Interactions with miRNAs

**Figure 2:**
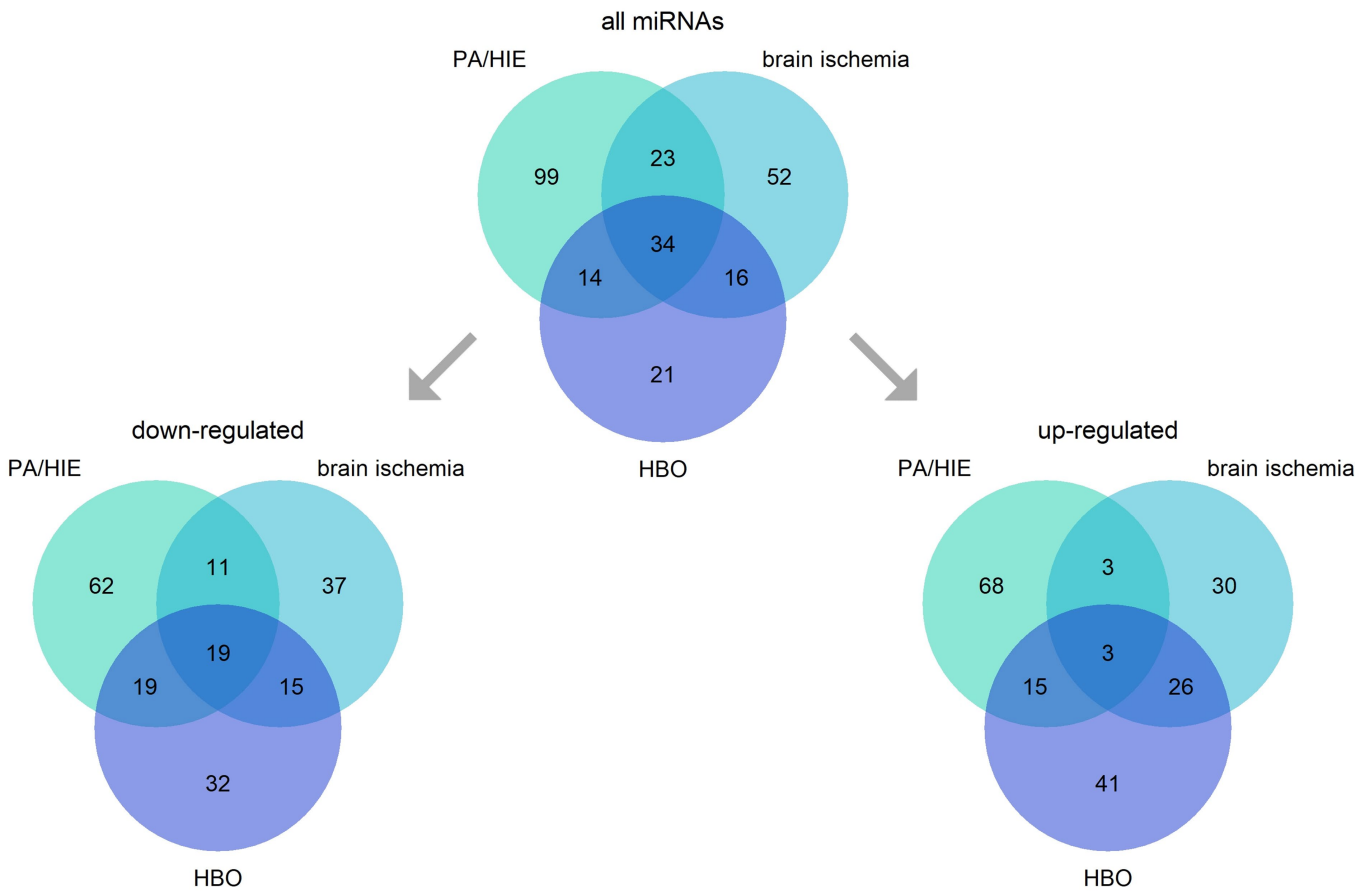
Intersections of miRNAs engaged in neonatal hypoxia-ischemia and its consequence —hypoxic–ischemic encephalopathy (PA/HIE), brain ischemia, and hyperbaric oxygen (HBO). The figure presents all miRNAs identified, and division into groups depending on their up-or down-regulation in analyzed conditions.

MicroRNAs (miRNAs) are small, ~22 nucleotide RNA molecules mainly responsible for the negative regulation of transcription of mRNAs either via their destabilization or translation blockade. Neurons are particularly enriched in microRNAs, with as much as half of them expressed in this cell type (44, 45). Recently, miRNAs have gained attention as potential diagnostic markers and therapy targets (46, 47). Research on perinatal HI and HIE identified a multitude of miRNAs involved in these processes, mainly regarding humans (umbilical cord blood—UCB and peripheral blood), mice, and piglets (UCB, carotid artery blood, and brain). Although direct reports on the involvement of miRNAs in HBO applied therapeutically upon birth asphyxia are lacking, we discuss this topic here. Several of the miRNAs identified as being important in neonatal HI, brain ischemia (middle carotid artery occlusion model) (48, 49), and HBO (including model of hyperoxia in retinopathy) are common for the all three (Figure 2).

Prominent among forms of brain injury after HI is cerebral white matter damage (WMD). HI-induced dysregulation of Wingless and Int-1(Wnt)/β-catenin signalling disrupts and delays normal myelination (50). HBOT administered within 12 h after HI alleviated WMD (51); while activating Wnt signaling (42). Many miRNAs regulate the Wnt pathways (canonical and non-canonical) at different levels (52–54). Both, mir-374a and mir-376c, altered in neonatal UCB of neonates upon suffering from HI and HIE, can regulate the Wnt/β-catenin pathway (55, 56). The non-canonical Wnt pathway is influenced by mir-128, mir-148a, mir-151a, mir-181a, and mir-181b, all upregulated in the blood of piglets subjected to HI (57, 58). Interestingly, mir-1264, upregulated in the mouse brain immediately after HI, can lead to apoptosis through Wnt/β-catenin signaling (59). The involvement of these miRNAs in the HBOT effect on Wnt signaling is highly probable.

The exposure of nucleus pulposus cells to HBOT (100% oxygen at 2.5 ATA) upregulated mir-107. This microRNA not only inhibits its direct target Wnt3a (60) but also suppresses the HMGB1/RAGE/NF-κB pathway, thus deactivating metalloproteases (MMP-3 and MMP-9), calcium-dependent zinc-containing endopeptidases that contribute to the disruption of the blood–brain barrier and extracellular matrix (61). Mir-107 was downregulated in UCB of human patients suffering from HI (56), as well as downregulated in blood and upregulated in the brains of rats subjected to transient focal cerebral ischemia (48); however, other studies found the opposite direction of changes (62, 63).

Another candidate microRNA to regulate the Wnt/β-catenin pathway, mir-185, is downregulated in UCB upon neonatal asphyxia (56), in the blood of rats subjected to brain ischemia (48), and in the blood of young stroke patients (62); however, mir-185 was upregulated when measured in whole brains (48). Notably, mir-185 inhibits glutathione peroxidase (GPx), an enzyme crucial in the antioxidant cell defense (64). Mir-107 also can regulate MAPK and subsequently NF-κB, thus influencing the inflammatory processes in the cells (65).

Many miRNAs can influence the activity of inflammation pathways; NF-κB impacts the expression of many miRNAs (66). MiRNAs altered upon HI that are central for the regulation of the Wnt/β-catenin pathway can also affect the activity of pro-inflammatory MAPK/NF-κB, including mir-331-5p downregulated in UCB after HI, mir-2137, and mir-137 upregulated after 24 h in mouse brain exposed to HI (59, 67, 68).

Li and co-workers reported that proteins expressed differentially in rat spinal cord injury upon HBOT were linked to mir-9a-5p and mir-125b-5p (69). Mir-9a-5p induced recovery after spinal cord ischemia–reperfusion injury through Notch signaling which is critical for developing and maintaining tissue homeostasis (70), while mir-125b inhibited SOD (64). Interestingly, mir-125b-5p was downregulated 48 h after birth in the venous blood in moderate/severe HIE with poor outcome (68) but upregulated in the brains of rats subjected to cerebral ischemia (48).

MiRNAs are also involved in the process of apoptosis after HI, including those engaging the Wnt pathway and inflammation processes (71). Those include mir-7, mir-128a, or mir-155 (72, 73). Some miRNAs involved in apoptosis were differentially expressed in UCB when comparing the severity of HIE, e.g., mir-98-5p was downregulated and mir-145-5p significantly upregulated in moderate/severe HIE with poor outcome as compared to no/mild HIE (59). Importantly, miRNAs influencing the activity of the Wnt pathway, e.g., mir-128, mir-148, mir-151a, mir-181a, mir-181b, mir-374a, or mir-376c-3p, can also regulate apoptosis (55, 58, 67). Mir-573, upregulated after HBOT, was responsible for the repression of Bax mRNA, consequently cleaved forms of caspase-3 and caspase-9 were also decreased (74).

The induction of the long non-coding RNA MALAT1 by HBO in human coronary artery endothelial cells and a rat model of acute myocardial infarction downregulated anti-angiogenic mir-15a and mir-92a (75, 76). Surprisingly, the mir-15a was downregulated in UCB of infants suffering from HI (56) but upregulated, along with mir-92a, in young stroke patients (62). The mir-155 was upregulated in the brains of mice 72 h after hypoxic–ischemic brain injury (67) and in rats 24 h after traumatic brain injury (63). Mir-155, which influences angiogenesis by repressing cysteine-rich angiogenic inducer 61 (CYR81, CCN1), produced in vascular cells and involved in redox homeostasis by inhibiting NOS and transcription factor ELK3, was upregulated in the brains of mice 72 h after HI brain injury (64, 67, 77, 78).

MiRNAs targeting hypoxia-inducible factor 1-alpha (HIF1α), specifically mir-137 and mir-335, were found to be upregulated 24 h and then downregulated 72 h after HI in mouse brains (67). Interestingly, mir-137 can inhibit NADPH oxidase (NOX), while mir-335 regulates NOS and SOD expression (64), thus being involved in redox homeostasis in cells. Other miRNAs essential for downregulating hypoxic response and downregulated in UCB, include mir-210, which has the potential to discern the severity of HIE, and mir-181b, which was significantly lowered in moderate and severe HIE (79). Thus, the involvement of these miRNAs in the HBOT effects is likely, but further research will be required.

### Combining HBOT with other therapies

Most studies conducted on experimental HI, as well as in reported clinical cases of neonatal HI, in which HBO therapy was applied, showed the beneficial effects of its use. However, researchers aiming to increase the effect combine HBO therapy with neuroprotective drugs used in clinical practice.

Memantine is a non-competitive, low-affinity blocker of NMDA receptors, and inhibiting these receptors has been considered a valid therapeutic approach for many years. In clinics, memantine is used to treat Alzheimer’s and Parkinson’s diseases; therefore, its combination with HBO in HI therapy seemed to be promising. The results presented by Gamdzyk and coworkers failed to demonstrate an additive increase in neuroprotection in experimental HI on 7-day-old rats upon combining HBOT with memantine 1 h or 6 h after HI insult (31). On the contrary, combining the treatments resulted in lower neuroprotection than the effects of HBO or memantine applied alone. However, Wang and coworkers reported that a combination of HBO and memantine treatments not only significantly reduced brain damage, oxidative stress, and inflammation in the brains of adult rats subjected to transient focal cerebral ischemia but also prolonged the therapeutic window from 6 to 12 h after reperfusion (80). These conflicting results may arise from differences between the expression of NMDA receptor subunits in developing and adult brains (31, 81, 82).

Ephedrine is a well-known sympathomimetic agent and a traditional Chinese herbal medicine and has been used to treat asthma, obesity, and narcolepsy. It has also been used to treat or prevent hypotension. The mechanism of ephedrine action relies on increasing the stimulation of the postsynaptic α and β adrenergic receptors by norepinephrine (83). Ephedrine crosses the blood–brain barrier and stimulates the nervous system, and some amphetamine-and methamphetamine-like responses were attributed to ephedrine. However, its medical use in small doses is accepted to treat several medical conditions. Ephedrine was also proposed as a stimulant to enhance the endurance and stamina of military service members (84). Chen and coworkers reported the neuroprotective effect of ephedrine alone and with HBO in newborn rats subjected to HI (85). In the experimental group treated with an ephedrine/HBOT combination, decreased brain edema and neuronal loss, inhibition of apoptosis, and improvement in memory formation were apparent when compared to controls and single-agent treatment. The results indicated that the combination had a synergistic effect; however, this combined therapy’s precise molecular and cellular mechanisms require further investigation.

Earlier publications indicated that monosialogangliosides significantly reduced white matter damage in experimental ischemia conducted in rats (86). It was also reported that in the clinical treatment of newborns suffering from HIE monosialogangliosides reduced serum apoptotic factors and increased serum content of SOD, GPx, brain-derived neurotrophic factor (BDNF), and nerve growth factor (NGF) while maintaining the stability of the internal environment (87). The use of monosialogangliosides as adjuvant treatment had also beneficial effects in the improvement of neurological outcomes (88). Interestingly, a meta-analysis performed by Gong and coworkers on using HBOT in clinical practice as adjuvant therapy for neonatal HIE mentioned 26 successful cases of combining HBOT with gangliosides (89).

Interestingly, the combination of HBO with erythropoietin in experimental rat spinal cord injury (90) and in humans diagnosed with cardiac insufficiency (91) offered promising results on combining HBO treatment with other agents.

## The clinical application of HBO therapy

HBOT is currently recommended for treating 14 conditions, including carbon monoxide poisoning, air embolism, diabetic foot, severe anemia, and decompression sickness (92). These treatments are well-accepted in adult patients and approved by the Undersea and Hyperbaric Medical Society (93). However, therapy for neonatal and pediatric patients is still treated with great reserve.

The first report on the use of HBO in the treatment of neonatal HI appeared in 1963. Hutchison and coworkers described that 35/65 (54%) children survived thanks to HBO therapy (8). However, this report was significantly criticized, and the authors were accused of using risky, not thoroughly proven therapy with insufficient preliminary animal research (7, 94).

Since then, the research into the use of HBOT in brain ischemia has progressed and a large volume of data has been published indicating its neuroprotective effects, including reports on the neuroprotective effects of HBOT in animal models of birth asphyxia. Thus, reports on the beneficial effects of HBO in brain ischemia, performed on animal models and clinical trials, have encouraged medical professionals in some countries to apply it in clinical practice. Scientists from the former USSR in the early 1980s reported almost 2,000 cases of the effective use of HBOT in the treatment of newborns suffering from birth asphyxia (Proceedings of VIIth International Congress of Hyperbaric Medicine, 1981). Articles published in Russian by Kostin, Baiborodov, and co-workers (37, 95–97) indicated the continuous use of HBOT in the clinic. Some reports from Mexico described the successful use of HBOT in treating term neonates with moderated HIE (98) and neonates with HIE and necrotizing enterocolitis (99). Despite the visible clinical improvement and no signs of hyperbaric oxygen side effects, the authors indicated technical problems due to the lack of intravenous pumps capable of working in hyperbaric conditions.

Nevertheless, technical progress has allowed the construction of large hyperbaric chambers that can accommodate a patient and qualified medical personnel. There are also specialized intravenous pumps and respirators adapted to work in hyperbaric conditions for newborns available on the market. Patients can also be monitored with ECG and with transcutaneous oxygen monitor (100). Moreover, for the additional safety of patients, including neonates, some countries have introduced specialized courses for nurses working with hyperbaric chambers. For example, the first hyperbaric nurses in the United States were certified in the late 1980s (101).

Apparently, HBOT is currently used mainly in China. Liu and coworkers published an extensive review article in which they discussed 20 trials, mainly from Chinese sources, in which HBOT at various doses, and in several cases with additional treatments, was used to treat hypoxic–ischemic neonates (100). The information presented in this review indicates that, although the use of HBOT in China is still controversial, this treatment reduced mortality and neurological sequelae in term neonates with HIE. Fifteen years later, Gong and coworkers published a systematic meta-analysis analyzing the use of HBOT as adjuvant therapy for neonatal HIE (89). They analyzed 46 selected randomized controlled clinical trials, including 4,199 patients with neonatal HIE. All of the trials were performed in China. Studies accomplished as part of this meta-analysis indicated that patients from the HBOT group exhibited a significantly better response to treatment than the control group. The incidence of sequelae was reduced, and the neonatal behavioral neurological assessment (NBNA) score of the HBOT group was significantly higher than that of untreated children. This meta-analysis also highlighted the lack of systematization of HBOT regarding pressure, exposure time, and duration of therapy. However, using a subgroup analysis, the authors determined the best treatment protocol for HBOT of neonates with HIE: (1) HBOT pressures 1.4–1.6 ATA are safe and effective for neonates suffering from HIE; (2) daily oxygen intake for 30–40 min provides the maximum therapeutic effect for neonates with HIE; and (3) the best therapeutic effect is achieved when the therapy lasts over 30 days. This is the first step towards creating an acceptable procedure for HBOT in the clinic, but given that the clinical trials were conducted in only one country, further larger studies are necessary.

## Discussion

Brain ischemia directly links to a plethora of pathologies, including stroke, cardiorespiratory arrest, and birth asphyxia. As it involves a reduced supply of oxygen to the brain, tissue hypoxia triggers vital molecular pathways such as excitotoxicity, oxidative stress, apoptosis, and inflammation that inevitably lead to cell death. Elevating oxygen levels in tissue affected by ischemia thus seems to be a valid approach to preventing neurodegeneration. HBOT is the most effective method of increasing dissolved oxygen concentration in plasma, providing the necessary oxygen supply to ischemic tissue. Breathing oxygen at a pressure of 2.8 ATA increases hemoglobin oxygenation (from 83 to 88%), cerebral blood flow, and regional cerebral oxygenation (102–104). Such conditions can counterbalance the HI-induced changes in the brain. Plentiful evidence obtained from animal studies and clinical trials indicates the beneficial, neuroprotective effects of HBOT used in the treatment of stroke, and traumatic brain injury, and its beneficial effect on ischemia–reperfusion injury (105–108). The authors reported the neuroprotective effect of HBOT resulting in metabolic and molecular rearrangements manifested by improved mitochondrial functioning along with a decrease in oxidative stress (30), acidosis and apoptosis inhibition, anti-inflammatory effects (109), coinciding with improved blood–brain barrier integrity, and reduced platelet aggregation and brain edema (110). Unfortunately, similar reports regarding experimental birth asphyxia, including clinical trials, are scarce. Available evidence indicates that HBOT applied within a short window after the HI reduces brain damage in newborns (16, 17, 31, 38, 89, 100).

Details of molecular mechanisms of neuroprotective HBOT effects come mainly from limited publications on animal models of neonatal HI, focusing on alterations caused by cerebral hypoxia or the antioxidant factors in the newborn blood. These reports show that HBOT significantly reduces HI-mediated ROS production, increases antioxidant enzyme activity, and inhibits apoptosis by restoring the balance between pro-and anti-apoptotic proteins in newborns’ brains and blood (Table 1). The activation of neuronal stem cells after HBOT reported in several publications also indicates this therapy’s considerable potential in preventing HI-evoked brain injury.

**Table 1:** Compilation of data showing the effect of HBOT used in HI on oxidative stress, apoptosis, and stem cell proliferation.

Affected process	Source	Pressure/time of exposition	Observed effects	References
Antioxidant defense	7-days old rat brains	2.5 ATA, 1,3 or 6 h after HI, 60 min	↓ ROS	Gamdzyk et al. (31)
	7-days old rat brains	2 ATA, 24 h after HI, 60 min/day, 14 days	↑SOD, ↓MDA	Chen et al. (32)
	Human neonates blood serum	1.4–1.6 ATA,?	↑SOD, ↓MDA, ↓NOS, ↓NO	Zhou et al. (36)
	Human neonates blood serum	?	↓ ROS, SOD no changes, ↑ GPx	Baiborodov et al. (97)
Apoptosis	7-days old rat brains	2.5 ATA, 1 h after HI, 90 min.	↓ caspase-3, ↓ AIF, ↓TUNEL positive cells	Liu et al. (39)
	7-days old rat brains	3 ATA, 1 h after HI, 60 min.	↓ caspase-3, ↓ PARP cleavage, ↓ TUNEL positive cells	Calvert et al. (38)
	7-days old rat brains	2.5 ATA, 1, 3 or 6 h after HI, 60 min.	↓ TUNEL positive cells	Gamdzyk et al. (31)
Stem cells proliferation	Neonatal rat brains	2 ATA, 3 h after HI, 60 min/day, 7 days	↑ newly generated neurons	Yang et al. (111)
	7-days old rat brains	2 ATA 3 h after HI, 60 min/day, 7 days	↑ migration of neural stem cells to the cortex	Wang et al. (112)
	7-days old rat brains	2 ATA 3 h after HI, 60 min/day, 7 days	↑ Wnt-3, nestin	Wang et al. (42)

The expression of many miRNAs is affected by HI or HBOT; however, data describing the effect of HBOT applied upon HI on miRNA expression are few. The significant diagnostic and therapeutic potential of miRNAs in many neurological disorders, including perinatal asphyxia and HIE, was strengthened by the recent findings on HBOT. The multitude and variety of the models and protocols used to collect samples diminishes the consistency of results. Multiple miRNAs can influence one biological process, further muddling the picture. The involvement of miRNAs in HBOT-mediated neuroprotection in birth asphyxia deserves further investigation, considering their use as potential biomarkers and therapeutic agents/targets.

In the clinical studies described, in which HBOT was used, clinical improvement in the condition of newborns suffering from HI was assessed using multiple methods, such as ECG and transcutaneous oxygen monitoring during HBO session, outcome indicators such as total efficiency (clinical symptoms, signs, and craniocerebral computed tomography), neuronal sequelae, and the NABA score (89, 98). The accumulated results of these studies indicate that the benefits of HBOT are obvious.

The consensus is that the primary source of beneficial effects of HBOT is an increase in the level of oxygen contained in the blood, especially oxygen dissolved in blood plasma. Indeed, breathing 100% oxygen at a pressure of 2 ATA increases the oxygen diffusion radius up to more than 4-fold. Consequently, the tissue oxygen concentration in the ischemic areas also increases (113).

Increased oxygen partial pressure causes hypothermia, which is desirable in the treatment of HI-affected neonates. Observed hypothermia was only partially the effect of pressure alone (114). However, a reduction of the temperature in the brains of gerbils subjected to ischemia was observed in animals treated with hyperbaric air (HBA), and in animals breathing 100% oxygen (NBO). Only a slight temperature drop was registered, although the oxygen partial pressure during NBO is two times higher than during HBA (115). Moreover, Fenton and colleagues observed a decrease in body temperature in experiments, where the partial pressure of oxygen in the air at 4 ATA was the same as the partial pressure in the air at 1 ATA (114). These experiments open new questions regarding the mechanism of action of HBOT.

### Summary

Considering the multifaceted HBOT effects that can alter multiple HI-instigated molecular processes resulting in neurodegeneration, there is clearly a need for more comprehensive research on the subject. High-quality, randomized clinical trials demonstrating the effectiveness of HBOT in neonates suffering from birth asphyxia will allow including it into clinicians’ therapeutic arsenal while shedding new light on the mechanistic intricacies of the approach.

## Author contributions

DM: Conceptualization, Visualization, Writing – original draft, Writing – review & editing. JG: Writing – review & editing. ES: Conceptualization, Supervision, Visualization, Writing – original draft, Writing – review & editing.
